# ATP Bioluminescence Assay To Evaluate Antibiotic Combinations against Extensively Drug-Resistant (XDR) Pseudomonas aeruginosa

**DOI:** 10.1128/spectrum.00651-22

**Published:** 2022-07-25

**Authors:** Berta Puig-Collderram, Sandra Domene-Ochoa, Maria Salvà-Comas, Maria Milagro Montero, Xavier Duran, Juan R. González, Santiago Grau, Antonio Oliver, Juan P. Horcajada, Eduardo Padilla, Concepción Segura, Núria Prim

**Affiliations:** a Laboratori de Referència de Catalunya, Barcelona, Spain; b Infectious Diseases Service, Hospital del Mar, Infectious Pathology and Antimicrobials Research Group (IPAR), Institut Hospital del Mar d'Investigacions Mèdiques (IMIM), Barcelona, Spain; c Institut Hospital del Mar d'Investigacions Mèdiques (IMIM), Barcelona, Spain; d Barcelona Institute for Global Health, ISGlobal, Barcelona, Spain; e Universitat Pompeu Fabra (UPF), Barcelona, Spain; f CIBER Epidemiología y Salud Pública (CIBERESP), Barcelona, Spain; g Pharmacy Department, Hospital del Mar, Barcelona, Spain; h Service of Microbiology, Hospital Son Espases, Instituto de Investigación Sanitaria Illes Balears (IdISBa), Palma de Mallorca, Spain; Louis Stokes Cleveland VAMC

**Keywords:** ATP, antibiotic combination, bioluminescence, luminometer, *Pseudomonas aeruginosa*, synergy

## Abstract

Time-kill curves are used to study antibiotic combinations, but the colony count method to obtain the results is time-consuming. The aim of the study was to validate an ATP assay as an alternative to the conventional colony count method in studies of antibiotic combinations. The cutoff point for synergy and bactericidal effect to categorize the results using this alternative method were determined in Pseudomonas aeruginosa. The ATP assay was performed using the GloMax 96 microplate luminometer (Promega), which measures bioluminescence in relative light units (RLU). To standardize this assay, background, linearity, and the detection limit were determined with one strain each of multidrug-resistant P. aeruginosa and Klebsiella pneumoniae. Twenty-four-hour time-kill curves were performed in parallel by both methods with 12 strains of P. aeruginosa. The conventional method was used as a “gold” standard to establish the pharmacodynamic cutoff points in the ATP method. Normal saline solution was established as washing/dilution medium. RLU signal correlated with CFU when the assay was performed within the linear range. The categorization of the pharmacodynamic parameters using the ATP assay was equivalent to that of the colony count method. The bactericidal effect and synergy cutoff points were 1.348 (93% sensitivity, 81% specificity) and 1.065 (95% sensitivity, 89% specificity) log RLU/mL, respectively. The ATP assay was useful to determine the effectiveness of antibiotic combinations in time-kill curves. This method, less laborious and faster than the colony count method, could be implemented in the clinical laboratory workflow.

**IMPORTANCE** Combining antibiotics is one of the few strategies available to overcome infections caused by multidrug-resistant bacteria. Time-kill curves are usually performed to evaluate antibiotic combinations, but obtaining results is too laborious to be routinely performed in a clinical laboratory. Our results support the utility of an ATP measurement assay using bioluminescence to determine the effectiveness of antibiotic combinations in time-kill curves. This method may be implemented in the clinical laboratory workflow as it is less laborious and faster than the conventional colony count method. Shortening the obtention of results to 24 h would also allow an earlier guided combined antibiotic treatment.

## INTRODUCTION

Use and abuse of antibiotics have led to an increase in antibiotic-resistant bacterial strains. Acquired resistance to antimicrobials is an issue challenging health systems. Antibiotic resistance can be caused by different mechanisms, ranging from inactivation or alteration of the antimicrobial or its binding site to changes in porins or in efflux pumps, which result in a lower accumulation of antibiotic. Infections caused by multidrug-resistant (MDR) microorganisms may be difficult to treat, which is translated into an increase in their mortality and morbidity rates.

The emergence of antibiotic resistance in bacteria usually involved in nosocomial infections, has generated special alarm because these infections can compromise the lives of critical and/or immunosuppressed patients. Pseudomonas aeruginosa and Klebsiella pneumoniae, together with Escherichia coli, are the bacterial species most frequently involved in nosocomial infections. These infections are often caused by MDR high-risk clones within these species (1–4).

Optimization of the strategies to combat MDR microorganisms is essential due to the limited therapeutic options. Combining antibiotics to obtain a synergistic effect against MDR bacteria may be also a therapeutic option. The efficacy of an antibiotic combination can be evaluated by different techniques. The time-kill curve is a dynamic method in which bacterial death is analyzed in the presence of fixed concentrations of antibiotic in relation to time (5). Results from time-kill curves are obtained 48 h after starting the experiment. Based on the results obtained in the curves, other complex pharmacokinetic/pharmacodynamic studies can be carried out, such as the “hollow fiber” model (6). This two-compartment model allows the evaluation of changes in antibiotic concentration over time, mimicking physiological conditions. Both techniques are very laborious and complicated when implemented in the clinical diagnostic routine in a microbiology laboratory. A less laborious and faster technique than the classical methodology to count viable cells would facilitate the laboratory workflow and shorten the time for results, allowing the implementation of studies of antibiotic combinations in the clinical routine.

Methods for bacterial detection and counting based on bioluminescence have been described. These methods correlate cell viability with metabolic activity by measuring NAD or ATP. The presence of these molecules in cells makes its detection symptomatic of the presence of living organisms (7).

Luminometers allow the quantification of bioluminescence. The increase in bioluminescence is directly proportional to the amount of ATP, which in turn correlates with the living organisms present in the sample (8). These systems are widely used, especially in industry and in health care settings, to evaluate the effectiveness of cleaning and/or sterilization processes (8, 9). The potential use of this technique to evaluate antibiotic activity by studying the bactericidal effect has been also reported (10). To our knowledge, the pharmacodynamic parameter of synergy has not been determined before using this technique in time-kill curves.

The aim of the present study was to validate the use of this ATP measurement assay using bioluminescence as an alternative to the colony count of viable cells for the study of antibiotic combinations. The ultimate goal is the implementation of this technique in the laboratory routine.

## RESULTS

### Luminometer standardization.

**FIG 1:**
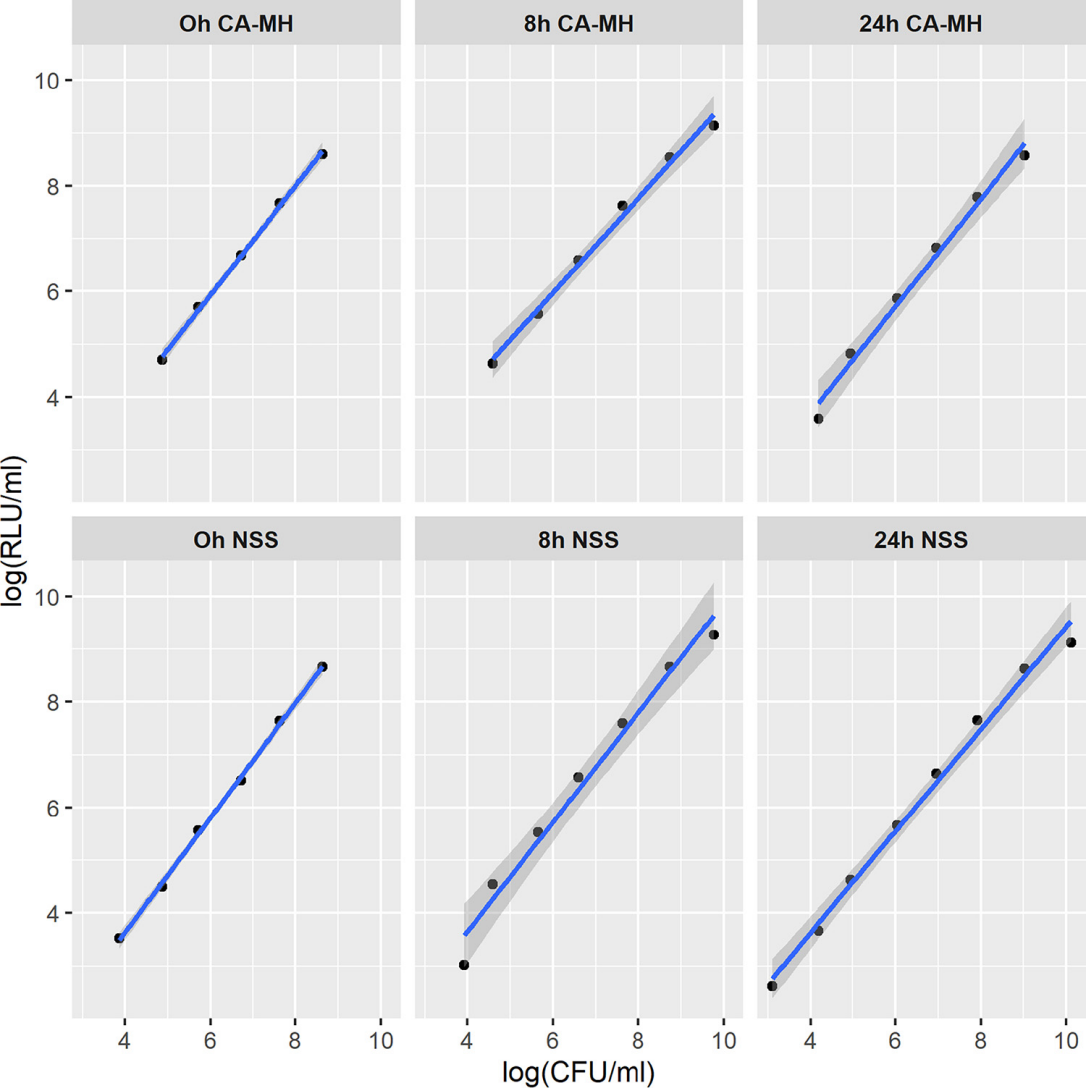
Linearity study of Pseudomonas aeruginosa at 0, 8, and 24 h with cation-adjusted Mueller-Hinton broth (CA-MHB) and normal saline solution (NSS) (below).

The background average values obtained from 30 observations were 10^3^ and 10^2^ relative light units (RLU)/mL for cation-adjusted Mueller-Hinton broth (CA-MHB) and normal saline solution (NSS), respectively. Below these values bacterial RLU readings could not be differentiated from the corresponding RLU value of the medium. Background oscillation was considered relevant enough to be removed in further assays. The detection limits obtained for both CA-MHB and NSS were 10^4^ and 10^3^ CFU/mL, respectively. The range of linear relationship between log RLU per milliliter and log CFU per milliliter were 10^4^ to 10^8^ CFU/mL (*R*^2^ = 0.99 to 1) in CA-MHB, and 10^3^ to 10^9^ CFU/mL (*R*^2^ = 0.98 to 1) in NSS (Fig. 1). No significant differences were observed at different times or for the different species. NSS was established in further studies as the medium used to wash and dilute the samples.

The relative standard deviations (RSDs), in both the CA-MHB and NSS data, were equal to or inferior to the corresponding RSDs of the conventional method for the different times, concentrations, and strains analyzed (data not shown).

### Comparative analysis.

The comparative analysis was performed analyzing the results from the P. aeruginosa curves by the conventional colony count method and the ATP assay in parallel. The growth curve study was performed from the values obtained under the experimental condition without antibiotic. The Pearson correlation coefficient (*r*) was 0.93, and the infraclass coefficient correlation (ICC) was 0.88 (95% confidence interval [CI], 0.816 to 0.923).

**FIG 2:**
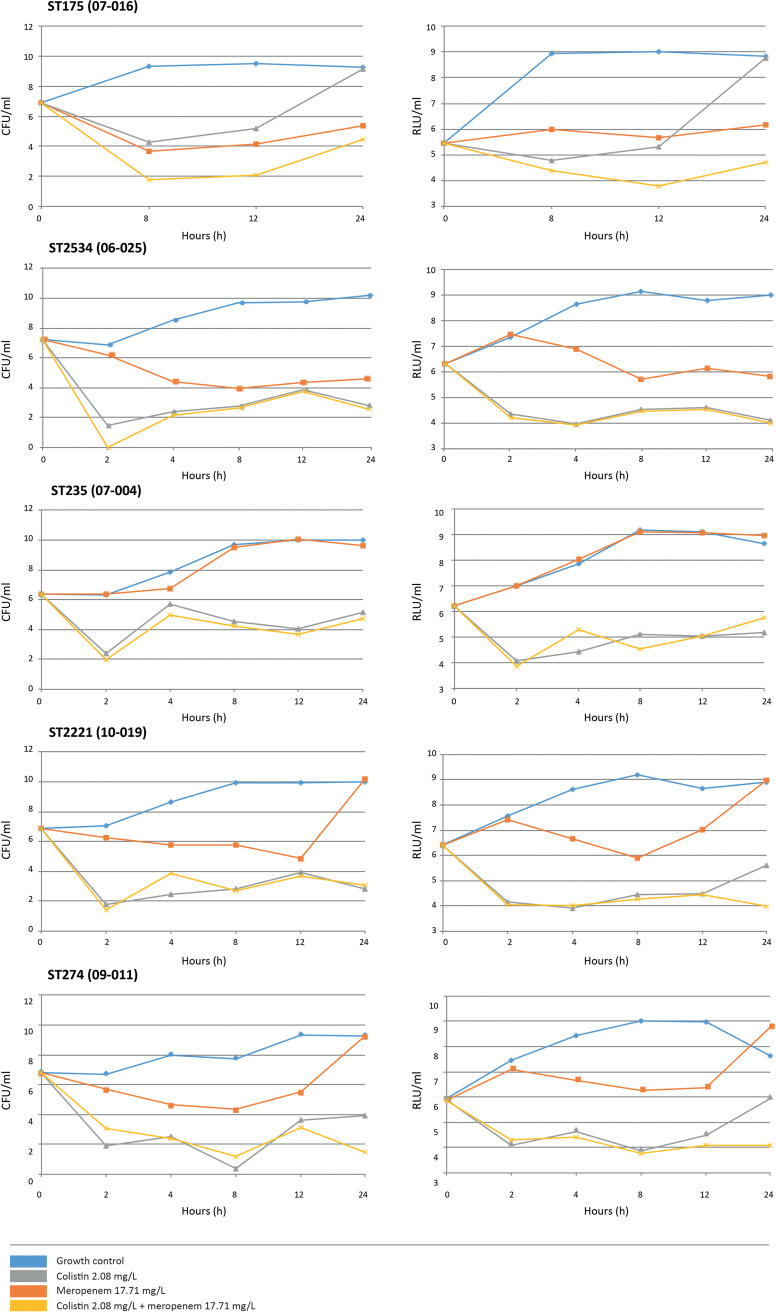
Time-kill curves for P. aeruginosa ST175 (07-016), ST235 (07-004), ST274 (09-011), ST2221 (10-016), ST2534 (06-025), and ST2536 (06-001). Results are shown for the conventional colony count method (CFU per milliliter) (left) and ATP measurement assay (RLU per milliliter) (right). Four experimental conditions were studied: growth control, meropenem alone, colistin alone, and meropenem plus colistin. The antibiotic concentrations were 17.71 mg/L for meropenem and 2.08 mg/L for colistin.

**FIG 3:**
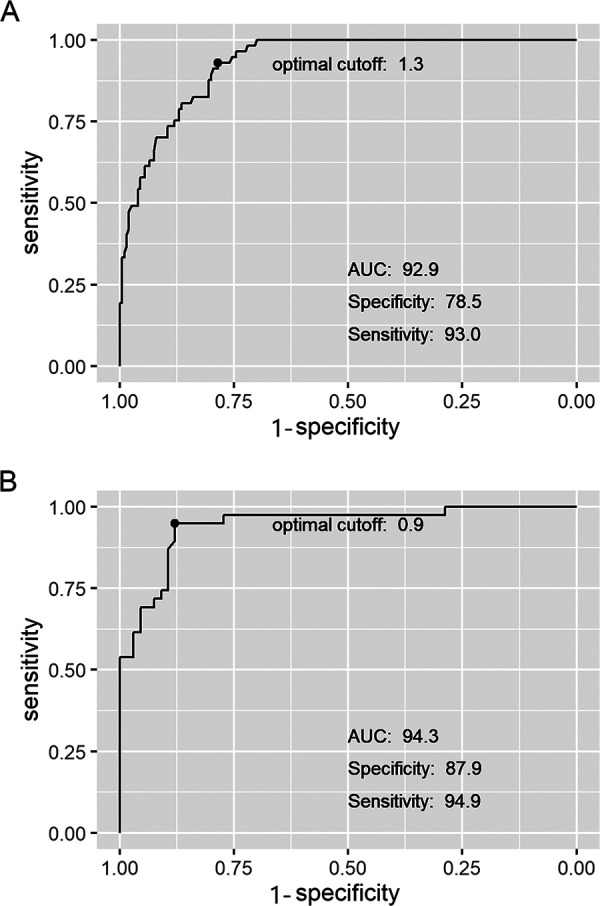
ROC curves for bactericidal effect and synergy. High areas under the ROC curves (0.929 to 0.943) were observed in both pharmacodynamic parameters bactericidal effect (A) and synergy (B).

The graphical comparison of time-kill curves for some isolates is shown in Fig. 2. The results obtained at 24 h by the conventional colony count method (CFU per milliliter) were used as a gold standard to determine the pharmacodynamic parameters in RLU per milliliter. The cutoff point for bactericidal effect was established using the values obtained from antibiotics as single agents and combined. The sample size included 257 paired data. The value for the bactericidal effect cutoff was 1.348 log RLU/mL, with a sensitivity of 93%, and a specificity of 78.5%; the area under the curve (AUC) obtained was 0.929 (Fig. 3A).

The cutoff point for synergy was established considering the values obtained from antibiotic combinations. The sample size included 105 paired data. The cutoff point for synergy was 0.9 log RLU/mL, with a sensitivity of 95% and a specificity of 88%; the area under the curve (AUC) obtained was 0.9430 (Fig. 3B). When considering a specificity of 100%, the cutoff was 2.199 (53% sensitivity).

## DISCUSSION

Combined antibiotic treatments are increasingly used in the clinic due to the burden of infections caused by MDR/extensively drug-resistant (XDR) bacteria. However, antibiotic combination studies are time-consuming and therefore not routinely performed in clinical laboratories. The use of instantaneous luminometer reading to evaluate antibiotic combinations in time-kill curves would avoid the requirement of an overnight culture for a viable cell count. The present study was aimed to validate an ATP measurement technique using bioluminescence as an alternative to the laborious conventional colony count method for the study of antibiotic combinations in time-kill curves.

Background, linearity, and the detection limit were studied for the standardization of the ATP measurement assay. NSS was compared with CA-MHB, which was the medium recommended by the manufacturer and used in previous studies (8, 11, 12). Background oscillation was considered relevant enough to be removed from assays. Linearity showed similar Pearson’s correlation coefficients. Given the lower detection limit and the broader range of linearity determined for NSS, this was established as the optimal medium. Considering the initial inoculum (10^6^ log CFU/mL), the detection limit obtained with NSS (10^3^ log CFU/mL) was considered adequate as the value corresponded to the maximum bacterial load change required to evaluate the pharmacodynamic parameters.

Control of variables affecting the bioluminescence reaction is essential, including the composition of the reading medium. Several ions have been reported to quench the bioluminescence reaction of the firefly luciferase (13). Cations should be strictly controlled while performing the assay, not only because of the potential quenching effect of the medium, but also due to the requirement of magnesium for the activity of this enzyme (11, 14). The concentration of cations included in CA-MHB medium may interfere with the RLU signal, which is in agreement with our standardization results that support the use of NSS instead of CA-MHB medium. Extracellular ATP may also affect the signal, as previously reported by Cai et al. (10). While these authors removed enzymatically extracellular ATP, the double centrifugation we performed to eliminate the antibiotic may have also contributed to its removal.

The correlation between CFU and RLU had been previous reported to diminish at 24 h, depending on the bacterial species (15). They attributed those discrepancies to metabolic differences between growth phases, slowing down the ATP production at the stationary phase. However, the decreases in correlations were observed because readings above the upper limit of what the authors had considered their linearity range were included. In contrast, in the present study no differences were found in the correlation at different growth phases—probably because the readings were within the linearity range.

The categorization of antibiotic combinations obtained using the ATP measurement assay was equivalent to the results obtained with the colony count method. Specificity should be prioritized over sensitivity when considering the therapeutic benefit of a combination. Therefore, 100% specificity cutoff points for synergy should be used to avoid false positives and unnecessary side effects. Synergy should be confirmed by the colony count method when the bacterial load decrease is between the cutoff with maximum sensitivity and specificity and the 100% specificity cutoff point. In this situation, the same aliquot could be further evaluated by the conventional method.

Our study had some limitations. The present study included only Pseudomonas aeruginosa. Other Gram-negative bacilli and Gram-positive bacteria should also be evaluated to validate its use in clinical isolates. One of the strengths of our study was the large sample size of readings to establish the cutoff points for P. aeruginosa. Another strength was the number of different antibiotic combinations, which supports its potential in a clinical background.

Our results support that the ATP assay could be used for the study of antibiotic combinations against MDR/XDR strains. Furthermore, as this method is less laborious and faster than the colony count, it could be implemented in the clinical laboratory workflow routine for the study of effective treatments against clinical isolates.

## MATERIALS AND METHODS

### Bacteria.

This study examined 12 clinical isolates of extensively drug-resistant (XDR) P. aeruginosa from nine Spanish hospitals previously characterized at a phenotypical and molecular level (see the supplemental material) (16). The isolates belonged to the following sequence types: ST111, ST175, ST179, ST235, ST244, ST253, ST274, ST2221, ST2533, ST2534, ST2535, and ST2536. KPC-containing K. pneumoniae ATCC BAA 1706 was included for standardization.

### Culture media and antibiotics.

Columbia blood agar (COS) (bioMérieux) were used to culture the isolates. Cation-adjusted Mueller-Hinton broth (CA-MHB) (Becton, Dickinson) was used to perform the time-kill curves, and Mueller-Hinton agar (MH) plates (bioMérieux) were used for the quantification of CFU per milliliter.

The antibiotics used were meropenem, aztreonam, colistin, and amikacin (all from Sigma-Aldrich), and ceftolozane-tazobactam (Zerbaxa; MSD). The antibiotic stocks were prepared according to the Clinical and Laboratory Standards Institute (CLSI). The final concentrations used in the time-kill assays corresponded to the AUC over 24 h (Table 1) (17–21).

**TABLE 1:** Antibiotics used in the time-kill curves^*a*^

Antibiotic	Concn (mg/L)	Equivalent clinical dose
Meropenem	17.71	2 g q8h
Aztreonam	43.75	2 g q8h
Ceftolozane-tazobactam	38/6.25	3 g q8h
Colistin	2.08	4.5 IU q12h
Amikacin	8.17	1 g q24h

a The corresponding concentration and its equivalent clinical dose are shown for each antibiotic.

### Time-kill curves.

A starter culture was performed from the overnight (O/N) culture in CA-MHB and incubated in a water bath shaker at 37°C for 90 min to achieve early exponential growth.

Each flask containing 30 mL of CA-MH was supplemented with either a single antibiotic or combination at the corresponding concentration (Table 1), except the growth control condition. The antibiotic combinations tested are shown in the supplemental material. Flasks were inoculated with 10^6^ CFU/mL and incubated at 37°C in the water bath shaker for 24 h. From each flask, 1-mL aliquots were collected at different times (0, 8, and 24 h) and centrifuged at 5,000 rpm for 5 min. After the supernatants were discarded, bacterial cells were resuspended twice with saline solution to eliminate the antibiotic from the medium. Each aliquot was analyzed in parallel using two methods: (i) conventional viable cell count (CFU per milliliter) and (ii) the ATP measurement assay (RLU per milliliter).

### Conventional viable cell count (CFU per milliliter).

Ten-fold dilutions of the aliquots were inoculated onto MH agar plates and incubated at 37°C for 18 to 24 h. Only plates with 30 to 300 colonies were considered.

The bactericidal effect and synergy were the pharmacodynamic parameters defined based on conventional viable cell count. The bactericidal effect is defined as the reduction of ≥3 log CFU/mL at 24 h from the start of the experiment with the starter culture (5). Synergy is defined as the reduction of ≥2 log CFU/mL from the final count of colonies in the antibiotic combination compared to the result of the most effective of the two components (22).

### ATP quantification technique by bioluminescence (RLU per milliliter).

The quantification of viable cells through ATP was performed using the BacTiter-Glo microbial cell viability assay kit (Promega) and the GloMax 96 microplate luminometer (Promega) (11). The reagent was prepared and preserved following manufacturer’s instructions. After bacterial lysis, free ATP reacts with luciferase enzyme (Ultra-Glo recombinant luciferase) that induces a luminescent signal. This assay is based on the ability of firefly luciferase to catalyze the oxidation of d-luciferin in the presence of a magnesium salt and ATP, and the light intensity emitted has been shown to be proportional to the ATP content and hence to the viable bacterial cells in any sample.

ATP measurement was performed using 96 opaque-walled multiwell plates. Time-kill curve samples were analyzed in triplicate and samples from the linearity assay five times. The same number of controls with background medium was included in each assay. The procedure was done by adding 100 μL sample and 100 μL BacTiter-Glo reagent. The plate was incubated at 37°C in the dark for 5 min and placed into the luminometer with a 1-s integration time. Mean background RLU values were then subtracted from the RLU sample values.

### ATP assay standardization.

Background, linearity, precision, and the limit of detection were analyzed using P. aeruginosa ST175 and K. pneumoniae ATCC BAA-1706 to set the experimental conditions to perform the time-kill curve tests. Each test of each strain was performed in duplicate.

Background was determined to control any significant oscillation of the reading medium. Data were gathered from linearity assays and corresponded to the values obtained with CA-MHB and normal saline solution (NSS) alone. The average and the standard deviation (SD) were calculated.

Linearity was calculated performing 10-fold dilutions of the bacterial culture in CA-MHB. This bacterial culture was performed as described for the time-kill curves. Two aliquots of each dilution were obtained at 0, 8, and 24 h to test two different backgrounds (i.e., CA-MHB and NSS). For the NSS condition, one aliquot was centrifuged at 5,000 rpm for 5 min, the supernatant was discarded, and the bacterial cells were resuspended twice with NSS. For the CA-MHB broth, there were no additional steps prior to the reading. Each aliquot was 10-fold diluted and analyzed in parallel using the luminometer and performing cultures onto MH plates in duplicate.

For every time and background, graphical representations of the results expressed in log_10_ scale were plotted. CFU were represented in the *x* axis and RLU in the *y* axis. Linear regression was used to evaluate the correlation between CFU and RLU using the *R*^2^ statistic.

Precision corresponded to the relative standard deviation (RSD) (coefficient of variation). This parameter was calculated for each time, viable cell count, and background (14).

The limit of detection was calculated from the control wells of the linearity assay as the average luminescence (RLU) + 3 SD (23).

### Statistical analyses.

The comparative study between the conventional method and the ATP assay included a correlation study throughout the growth curves. The average of time-kill curve results (CFU and RLU) was converted to a log_10_ scale. The values from the P. aeruginosa growth control (without antibiotic) were used to confirm the correlation between CFU and RLU along the 24-h growth curves. A Shapiro-Wilk test for normal data was performed prior to calculation of the Pearson correlation coefficient and the intraclass correlation coefficient (ICC) with Stata version 15.

Receiver operating characteristic (ROC) curves, sensitivity, specificity, area under the ROC curve (AUC), and the optimal cutoff for the ATP assay were computed using an R library called pROC. Figures were created in R using the ggplot library. The bactericidal effect and synergy cutoff points were determined with a Youden test (14) using the conventional colony count method as the gold standard. The chosen cutoff point values were those with maximum sensitivity and specificity. Nonetheless, the sensitivity and specificity of these parameters can be adjusted through the modification of the cutoff point values according to the aim of the test.

## References

[1] Oliver A, Mulet X, López-Causapé C, Juan C. 2015. The increasing threat of Pseudomonas aeruginosa high-risk clones. Drug Resist Updat 21–22:41–59. doi:10.1016/j.drup.2015.08.002. 26304792

[2] Mathers AJ, Peirano G, Pitout JDD. 2015. The role of epidemic resistance plasmids and international high-risk clones in the spread of multidrug-resistant Enterobacteriaceae. Clin Microbiol Rev 28:565–591. doi:10.1128/CMR.00116-14. 25926236 PMC4405625

[3] Breidenstein EBM, de la Fuente-Núñez C, Hancock REW. 2011. Pseudomonas aeruginosa: all roads lead to resistance. Trends Microbiol 19:419–426. doi:10.1016/j.tim.2011.04.005. 21664819

[4] Banerjee R, Johnson JR. 2014. A new clone sweeps clean: the enigmatic emergence of Escherichia coli sequence type 131. Antimicrob Agents Chemother 58:4997–5004. doi:10.1128/AAC.02824-14. 24867985 PMC4135879

[5] Leber AL (ed). 2016. Clinical microbiology procedures handbook, 4th ed. American Society for Microbiology, Washington, DC.

[6] Drusano GL, Neely MN, Yamada WM, Duncanson B, Brown D, Maynard M, Vicciarella M, Louie A. 2018. The combination of fosfomycin plus meropenem is synergistic for Pseudomonas aeruginosa PAO1 in a hollow-fiber infection model. Antimicrob Agents Chemother 62:e01682-18. doi:10.1128/AAC.01682-18. PMC6256766 30249700

[7] Shama G, Malik DJ. 2013. The uses and abuses of rapid bioluminescence-based ATP assays. Int J Hyg Environ Health 216:115–125. doi:10.1016/j.ijheh.2012.03.009. 22541898

[8] Cai Y, Leck H, Lim TP, Teo J, Lee W, Hsu LY, Koh TH, Tan TT, Tan T-Y, Kwa AL-H. 2015. Using an adenosine triphosphate bioluminescent assay to determine effective antibiotic combinations against carbapenem-resistant Gram negative bacteria within 24 hours. PLoS One 10:e0140446-15. doi:10.1371/journal.pone.0140446. 26460891 PMC4603788

[9] Aragonès L, Escudé C, Visa P, Salvi L, Mocé-Llivina L. 2012. New insights for rapid evaluation of bactericidal activity: a semi-automated bioluminescent ATP assay. J Appl Microbiol 113:114–125. doi:10.1111/j.1365-2672.2012.05320.x. 22530985

[10] Cai Y, Ng JJ, Leck H, Teo JQ, Goh J-X, Lee W, Koh T-H, Tan T-T, Lim T-P, Kwa AL. 2020. Elimination of extracellular adenosine triphosphate for the rapid prediction of quantitative plate counts in 24 h time-kill studies against carbapenem-resistant Gram-negative bacteria. Microorganisms 8:1489–1415. doi:10.3390/microorganisms8101489. 32998347 PMC7599598

[11] Promega. 2012. BacTiter-Glo microbial cell viability assay. 67. Promega, Madison, WI.

[12] Cai Y, Seah CL, Leck H, Lim T-P, Teo JQ, Lee W, Tan T-T, Koh T-H, Ee PLR, Kwa AL. 2018. Rapid antibiotic combination testing for carbapenem-resistant Gram-negative bacteria within six hours using ATP bioluminescence. Antimicrob Agents Chemother 62:e00183-18. doi:10.1128/AAC.00183-18. 29967021 PMC6125559

[13] Zhang H, Bai H, Jiang T, Ma Z, Cheng Y, Zhou Y, Du L, Li M. 2016. Quenching the firefly bioluminescence by various ions. Photochem Photobiol Sci 15:244–249. doi:10.1039/c5pp00432b. 26789132

[14] OIE. 2012. Principios y métodos de validación de las pruebas de diagnóstico de las enfermedades infecciosas. Man Acuático OIE 2012:20.

[15] Vogel SJ, Tank M, Goodyear N. 2014. Variation in detection limits between bacterial growth phases and precision of an ATP bioluminescence system. Lett Appl Microbiol 58:370–375. doi:10.1111/lam.12199. 24330032

[16] Del Barrio-Tofiño E, López-Causapé C, Cabot G, Rivera A, Benito N, Segura C, Montero MM, Sorlí L, Tubau F, Gómez-Zorrilla S, Tormo N, Durá-Navarro R, Viedma E, Resino-Foz E, Fernández-Martínez M, González-Rico C, Alejo-Cancho I, Martínez JA, Labayru-Echve OA. 2017. Genomics and susceptibility profiles of extensively drug-resistant Pseudomonas. Antimicrob Agents Chemother 61:e01589-17. doi:10.1128/AAC.01589-17. 28874376 PMC5655108

[17] Kitzes-Cohen R, Farin D, Piva G, De Myttenaere-Bursztein SA. 2002. Pharmacokinetics and pharmacodynamics of meropenem in critically ill patients. Int J Antimicrob Agents 19:105–110. doi:10.1016/S0924-8579(01)00474-5. 11850162

[18] Smith PF, Ballow CH, Booker BM, Forrest A, Schentag JJ. 2001. Pharmacokinetics and pharmacodynamics of aztreonam and tobramycin in hospitalized patients. Clin Ther 23:1231–1244. doi:10.1016/S0149-2918(01)80103-X. 11558860

[19] Wooley M, Miller B, Krishna G, Hershberger E, Chandorkar G. 2014. Impact of renal function on the pharmacokinetics and safety of ceftolozane-tazobactam. Antimicrob Agents Chemother 58:2249–2255. doi:10.1128/AAC.02151-13. 24492369 PMC4023800

[20] Marchand S, Lamarche I, Gobin P, Couet W. 2010. Dose-ranging pharmacokinetics of colistin methanesulphonate (CMS) and colistin in rats following single intravenous CMS doses. J Antimicrob Chemother 65:1753–1758. doi:10.1093/jac/dkq183. 20507861

[21] Garraffo R, Drugeon HB, Dellamonica P, Bernard E, Lapalus P. 1990. Determination of optimal dosage regimen for amikacin in healthy volunteers by study of pharmacokinetics and bactericidal activity. Antimicrob Agents Chemother 34:614–621. doi:10.1128/AAC.34.4.614. 2111658 PMC171653

[22] Lim T-P, Cai Y, Hong Y, Chan ECY, Suranthran S, Teo JQ-M, Lee WH, Tan T-Y, Hsu L-Y, Koh T-H, Tan T-T, Kwa AL-H. 2015. In vitro pharmacodynamics of various antibiotics in combination against extensively drug-resistant Klebsiella pneumoniae. Antimicrob Agents Chemother 59:2515–2524. doi:10.1128/AAC.03639-14. 25691628 PMC4394811

[23] Cáñez-Carrasco MG, García-Alegría AM. 2015. Validación de un método analítico para la determinación de fósforo por espectrofotometría ultravioleta-visible. BIOtechnia 17:32. doi:10.18633/bt.v17i1.15.

