# Supplementary material for: ATP Bioluminescence Assay To Evaluate Antibiotic Combinations against Extensively Drug-Resistant (XDR) Pseudomonas aeruginosa
Source: Microbiol Spectr. 2022 Jul 25;10(4):e00651-22. doi: 10.1128/spectrum.00651-22 (PMC9431428; doi:10.1128/spectrum.00651-22)
Supplement: Supplemental file 1 — Supplemental material. Download spectrum.00651-22-s0001.pdf, PDF file, 0.1 MB [file spectrum.00651-22-s0001.pdf]

SUPPLEMENTARY DATA

Appendix A:

***P. aeruginosa* isolate**	**ST**	**Mechanisms of resistance**
**10-009**	**111**	VIM-2, AmpC hyperproduction, OprD deficiency
**07-016**	**175**	GES-5, OprD deficiency
**10-023**	**175**	AmpC hyperproduction, OprD deficiency
**12-012**	**175**	VIM-20, OXA-2, OprD deficiency
**06-014**	**179**	OXA-10, AmpC hyperproduction, OprD deficiency
**07-004**	**235**	GES-19, OXA-2, OprD deficiency
**12-003**	**244**	AmpC hyperproduction, OprD deficiency
**01-008**	**253**	VIM-1, OprD deficiency
**09-011**	**274**	AmpC hyperproduction, OprD deficiency
**10-019**	**2221**	AmpC hyperproduction, OprD deficiency
**10-021**	**2533**	AmpC hyperproduction, OprD deficiency
**06-025**	**2534**	AmpC hyperproduction, OprD deficiency
**06-027**	**2535**	AmpC hyperproduction
**06-001**	**2536**	AmpC hyperproduction, OprD deficiency

Table. *Pseudomonas aeruginosa* isolates. The name of the isolate, the corresponding sequence type (ST) and the mechanisms of resistance are shown. Adapted from Oliver A. *et al.*(1)

Appendix B:

ANTIBIOTIC COMBINATIONS
Amikacin + meropenem
Aztreonam + ceftolozane/tazobactam
Aztreonam + amikacina
Aztreonam + colistin
Aztreonam + meropenem
Aztreonam + ceftolozane/tazobactam + colistin
Aztreonam + meropenem + colistin
Ceftolozane/tazobactam + colistin
Ceftolozane/tazobactam + amikacin
Ceftolozane/tazobactam + meropenem
Colistin + meropenem
Ceftolozane/tazobactam + colistin + meropenem

Table. Antibiotic combinations tested in time-kill curves. The antibiotic concentrations were: amikacin 8.17 mg/L, aztreonam 43.75mg/L, colistin 2.08 mg/L, ceftolozane/tazobactam 38/6.25 mg/L and meropenem 17.71 mg/L.
